# Mal-Practice Case

**Published:** 1879-04

**Authors:** 


### MAL-PRACTICE CASE.

A case of prosecution for mal-practice, in which a Miss Brooks, of Columbus, Ohio, was plaintiff, and Dr. Todd, D. D. S., of the same city, was defendant, has, after a trial lasting thirteen days, been decided adverse to the defendant, judgment being obtained against him for nine hundred and twenty-five dollars.

The operation involved in the case was an attempt to extract an inferior third molar, in which the tooth seems by some means to have escaped the grasp of the instrument used, and passed into the soft tissues on the inner side of the jaw near to its angle, and remainining there for several weeks; it was alleged to have been the cause of extensive disease in the parts, and great suffering and prostration. This, it is presumed, was proven in court.

The tooth, after several weeks lodgment, was removed by another operator.

Of the merits of this case I know but little, having never seen it, and have had only indefinite descriptions of it.

It is quite possible that the manipulation for the removal may have been faulty, or slight inattention on the part of the operator, may have been an element in the disaster, or there may have been no fault in either of these respects, and yet to the struggling and resistance of the patient, the accident may have been wholly due; to such an extent does this sometimes occur that the wonder is, that serious accidents do not more frequently take place.

In this instance it is certain that, from some cause or other, a firm and proper grasp was not made upon the tooth with a proper instrument, or it could not have escaped as it did.

This case contains another lesson to the members of our profession, viz: that they should better understand the legal relation that exists between them and their patients; that they should in all doubtful cases put themselves in a safe position before proceeding to operate.

Then again, it should be a stimulus to those seeking to enter the profession, to make themselves most thoroughly familiar with the best modes of practice, and be able to put them into execution.

There are very few, if any, in the practice of dentistry or surgery, who might not, through the machination of mischievous attorneys and the ill advice of friends, be entangled in the meshes of mal-practice suits, not perhaps in many instances with the results of the above case, but any such entanglement is disasterous, whatever the issue may be.

In every such case the members of the profession should come to the rescue, unless it be manifestly a case of mal-practice, and in some such cases money can not make sufficient restitution.

If mal-practice suits are encouraged upon every frivolous pretense, then no one knows when he is safe.

Let the profession demand of its own members the largest measure of attainments and ability, and then stand solid as one man against unjust prosecutions for alleged mal-practice.

